# *In Vitro* and *In Vivo* Anti-Allergic and Anti-Inflammatory Effects of eBV, a Newly Developed Derivative of Bee Venom, through Modulation of IRF3 Signaling Pathway in a Carrageenan-Induced Edema Model

**DOI:** 10.1371/journal.pone.0168120

**Published:** 2016-12-08

**Authors:** Hwa-Jin Chung, Jinho Lee, Joon-Shik Shin, Me-riong Kim, Wonil Koh, Min-Jeong Kim, Jae-woong Lee, Eun Jee Kim, In-Hee Lee, Won Kyung Kim, Yoon Jae Lee, Sang Kook Lee, In-Hyuk Ha

**Affiliations:** 1 Jaseng Spine and Joint Research Institute, Jaseng Medical Foundation, Seoul, Republic of Korea; 2 College of Pharmacy, Natural Products Research Institute, Seoul National University, Seoul, Republic of Korea; National University Singapore Yong Loo Lin School of Medicine, SINGAPORE

## Abstract

**Background:**

Bee venom (BV), a type of toxin extracted from honeybees (*Apis mellifera*), has been empirically and widely used to treat inflammatory diseases throughout Asia. Essential BV (eBV) was developed by removing phospholipase A2 (PLA2) and histamine to lower occurrence of allergic reaction. This study investigated the anti-allergic and anti-inflammatory activities of eBV *in vitro* and *in vivo* and its underlying mechanism of action.

**Methods:**

The anti-inflammatory potential of eBV was assessed *in vivo* using a carrageenan-induced paw edema model. To further investigate the mechanism by which eBV exerts anti-allergic and anti-inflammatory effects, compound 48/80-stimulated RBL-2H3 cells and lipopolysaccharide (LPS)-stimulated RAW 264.7 murine macrophage cells were studied *in vitro*.

**Results:**

Release of β-hexosaminidase and histamine was increased by eBV in a dose-dependent manner, but these levels were lower in eBV compared to original BV at the same concentration. In addition, eBV suppressed compound 48/80-induced expression of tumor necrosis factor-α (TNF-α) and interleukin-1β (IL-1β) in RBL-2H3 cells. eBV was also shown to suppress nitric oxide (NO) production by down-regulating mRNA expression and subsequent protein expression of inflammatory mediators in LPS-induced RAW 264.7 cells. Phosphorylation of activators and signal transducers of transcription 1/interferon regulatory factor 3 (STAT1/IRF3) was attenuated by eBV treatment. eBV significantly inhibited carrageenan-induced acute edema *in vivo*. Serum levels of prostaglandin E2 (PGE2), TNF-α, and IL-1β were also down-regulated by eBV.

**Conclusions:**

These results demonstrate that eBV inhibits allergic and inflammatory response by reducing inflammatory mediator production via regulation of the STAT1/IRF3 signaling pathway, suggesting that eBV is a feasible candidate for regulation of allergic-inflammatory response in complementary and alternative medicine.

## Introduction

Bee venom (BV) is the toxin secreted from ovipositors of honeybees (*Apis mellifera*). It has a density of 1.3, pH of 5.2, bitter taste, and weak aromatic character. Seventy-five percent of BV is made up of various peptides and proteins, i.e., melittin, apamin, mast cell degranulating (MCD) peptide, phospholipase A2 (PLA2), adolapine, hyaluronidase, and histidine [1, 2]. BV has been reported to be effective against arthritis [3], atherosclerosis [4], low back pain [5], scarring of skin [6], microbes [7], inflammation [8, 9], and whitening of skin and wrinkles [10]. Melittin, widely accepted to be the principal active component in BV, has been shown to possess analgesic [11] and anti-inflammatory effects [12], and comprises more than 50% of BV.

In Asia, acupuncture with BV has been traditionally practiced using the stinger of living bees to stimulate certain acupoints. However, several challenges have arisen in application of this intervention. In an attempt to overcome such challenges, the toxin in ovipositors of living bees is extracted through electric stimulation, then dried and purified. Reported properties of BV pharmacopuncture include anti-inflammatory [13] and analgesic effects [14], and modulation of the immune system [15], and BV pharmacopuncture has been reported to be effective against rheumatic arthritis [16], lumbar herniation of intervertebral disc [17, 18], and degenerative knee arthritis [19] in various clinical studies.

Despite its numerous benefits, the allergic reactions that have been reported to occur during BV treatment remain a burden for both physicians and patients. Especially immediate systemic hypersensitivity, also known as anaphylactic shock, which presents in individuals allergic to BV, poses a rare but substantial threat. Substances known to be primarily responsible for allergic reactions are PLA2 and histamine, which entail safety concerns for BV-sensitive patients [20, 21].

The term *allergy* refers to a hypersensitive immunologic reaction to otherwise non-harmful antigens (known as *allergens*) that occur when the immune system is dysfunctional from either congenital or acquired causes. Upon inflammatory or allergic response, activated macrophages present antigens and secrete inflammatory cytokines such as TNF-α, IL-4, MCP-1, IL-8, and IL-13. These cytokines lead to inflammatory response through differentiation and maturation of other pro-inflammatory cells and synthesis of immunoglobulin, resulting in further allergic inflammation [22, 23].

Inflammation is a physiological self-defense mechanism that is triggered through systemic or local damage caused by such factors as infection, pathogens, or stress [24], and is generally classified as either acute or chronic.

During the inflammatory process, LPS binds to the complex of Toll-like receptor-4 (TLR4) and myeloid differentiation protein-2 (MD-2), thus inducing inflammatory response in macrophages [25]. Activated TLR4 then transmits downstream signals through the myeloid differentiation factor-88 (Myd-88)-dependent pathway and TIR-domain-containing adaptor protein, inducing activation of the IFN-β (TRIF)-dependent pathway. In the TRIF-dependent pathway, phosphorylated interferon regulatory factor 3 (IRF3) translocates to the nucleus and binds to the promoter region, up-regulating mRNA expression of type 1 IFN. Consequently, type 1 IFN binds to Janus kinases (JAKs) and phosphorylates signal transducer and activator of transcription-1 (STAT1), and phosphorylated STAT1, in turn, translocates to the nucleus and acts as a transcription factor promoting expression of such inflammatory factors as NO and PG [26, 27]. Therefore, modulation of such pathways through suppression of specific inflammatory mediators may be considered in treatment approaches to inflammatory disease [28].

In the present study, essential BV (eBV) was developed by removing PLA2 and histamine to ensure safe use of refined BV and prevent against adverse effects.

## Materials and Methods

### Chemicals

Dried BV was commercially purchased (Chung Jin Biotech Co. Ltd., Ansan, Korea). For quantitative analyses, water, acetonitrile (ACN), and methanol (MeOH) of high performance liquid chromatography (HPLC) grades were purchased from JT Baker (Phillipsburg, NJ, USA). Trifluoroacetic acid (TFA) was purchased from Junsei Chemical (Tokyo, Japan). Dulbecco’s modified Eagle’s medium (DMEM), fetal bovine serum (FBS), sodium pyruvate, L-glutamine, antimycotics-antibiotics solution, and trypsin-EDTA were purchased from Invitrogen Co. (Grand Island, NY, USA). Goat anti-rabbit IgG-HRP, goat anti-mouse IgG-HRP, goat anti-goat IgG-HRP, and primary antibodies against iNOS, COX-2, TNF-α, IL-1β, p65, p-p65, IκB-α, and β-actin proteins were purchased from Santa Cruz Biotechnology Inc. (Santa Cruz, CA, USA), and antibodies against IRF3, p-IRF3, STAT1, p-STAT1, STAT3, and p-STAT3 were bought from Cell Signaling Technology (Beverly, MA, USA). Oligonucleotides for PCR primers were synthesized by Bioneer (Daejeon, Korea). AMV reverse transcriptase, dNTP mixture, random primers, RNasin, and *Taq* polymerase were bought from Promega (Madison, WI, USA). Lipopolysaccharide (LPS, E. coli 0111:B4), melittin, polyinosinic–polycytidylic acid [poly(I:C)], 3-(4,5-dimethylthiazol-2-yl)-2,5-diphenyltetrazolium bromide (MTT), sulfanilamide, N-(1-naphthyl)-ethylenediamine dihydrochloride, dimethyl sulfoxide (DMSO) and reagents not otherwise specified were purchased from Sigma-Aldrich (St. Louis, MO, USA).

### Preparation of eBV

Ten grams of dried BV was dissolved in 20 mL of filtered water for sample purification. After a sephadex G-25 column (diameter 6 × 90 cm) was thoroughly rinsed with filtered water, the loaded sample with elution was separated into serial fractions of 40 mL each. Each fraction was then analyzed using LC/MS system (LCMS-2020, Shimadzu, Kyoto, Japan), and fractions removed of histamine were collected (histamine-free fractions). The collected fractions were then filtered using Cogent M1 TFF system (Merck, Darmstadt, Germany) to discard all molecules with molecular weight equal or greater than 10 kDa such as PLA2 (PLA2-free fractions). The final output was freeze-dried and stored at -20°C.

### HPLC fingerprinting of eBV

Standards of melittin, PLA2, and histamine were dissolved in HPLC-grade water to yield concentrations of 0.53 mg/mL, 1 mg/mL, and 0.01 mg/mL, respectively. Stock samples of non-refined BV and refined eBV were each brought to a concentration of 1 mg/mL for analysis.

To perform melittin and PLA2 fingerprinting of eBV using the LC/MS system, an injection volume of 10 μL was passed through a TC-C18 column (5 μm, 4.6 I.D. × 150 mm, Agilent); the column temperature was set at 35°C, and the flow rate was maintained at 0.4 mL/min. As the mobile phase, 0.1% TFA-distilled water and 0.1% TFA-ACN were used after degassing by ultrasonication, and analyses were conducted according to a gradient system (95:5, 0→10 min / 36:64, 10→19 min / 95:5, 19→30 min). For histamine fingerprinting, an injection volume of 5 μL was passed through a hilic silica column (3 μm, 2.1 I.D. × 100 mm, Agilent); the column temperature was set to 45°C, and the flow rate was maintained at 0.4 mL/min. As mobile phase, 100 mM ammonium formate (pH 3.0) and ACN were used after degassing by ultrasonication, and analysis was conducted based on a gradient system (10:90, 0→1 min / 50:50, 1→3.5 min / 10:90, 3.5→7 min). A UV detector with wavelength of 220 nm was used for detection of melittin and PLA2, and an MS detector (SIM (+), *m/z* 112.2) was used to determine histamine amount.

### Cell culture

RAW 264.7 cells originating from murine macrophages and RBL-2H3 cells from rat basophilic leukemia were obtained from American Type Culture Collection (ATCC, Manassas, VA, USA). Both RAW 264.7 cells and RBL-2H3 cells were cultured in DMEM supplemented with 10% FBS and antibiotics-antimycotics (100 U/mL penicillin G sodium, 100 μg/mL streptomycin sulfate, and 0.25 μg/mL amphotericin B). All cells were incubated in a 5% CO_2_ incubator at 37°C and were sub-cultured 3 ~ 4 times a week.

### β-Hexosaminidase degranulation assay

To investigate the suppressive effect of eBV on degranulation, a sign of type 1 immediate allergic response, secretion of β-hexosaminidase was analyzed. Following suspension in 10% FBS-supplemented DMEM, RBL-2H3 cells were seeded in 48-well plates (Corning Incorporated, Corning, NY, USA) at a density of 5 × 10^5^ cells/mL and incubated for 48 h. After washing twice with Tyrode’s buffer (137 mM NaCl, 2.7 mM KCl, 1.8 mM CaCl_2_, 1.1 mM MgCl_2_, 11.9 mM NaHCO_3_, 0.4 mM NaH_2_PO_4_, and 5.6 mM glucose, pH 7.2), each well was treated with various concentrations of eBV for 30 min at 37°C. Then, compound 48/80 (Sigma-Aldrich, St. Louis, MO, USA) was added to each well to reach a concentration of 5 mg/mL, and cells were incubated for an additional 30 min under the same conditions. Afterwards, cells were placed in an ice-bath for 10 min to terminate incubation and centrifuged at 5,000 rpm for 10 min. Supernatants were collected, and 30 μL of each sample was added to an equal volume of substrate buffer (1 mM p-nitrophenyl-N-acetyl-β-D-glucosamine in 0.05 M citrate buffer, pH 4.5). Following 1 h of incubation, reaction was stopped with twice the volume of stopping buffer (0.1 M Na_2_CO_3_/NaHCO_3_, pH 10.0). Absorbance was read at 407 nm using a microplate reader (Molecular Devices Co. Ltd., Sunnyvale, CA, USA).

### Histamine degranulation assay

To assess release of histamine in RBL-2H3 cells, 1 μL of the supernatant that was obtained prior to β-hexosaminidase degranulation assay was added to 0.2 mL 1 N NaOH and 0.1 μL 1% o-phthalaldehyde (OPA). The mixture was allowed to rest at room temperature for 5 min. After stopping the reaction with 0.2 mL 1 N HCl, histamine content was examined using LC/MS.

### TNF-α and IL-4 assay

The released amounts of TNF-α and IL-4 cytokines in supernatant and mRNA expression were determined. The secretion levels of TNF-α and IL-4 were examined in supernatants collected from the β-hexosaminidase degranulation assay, which were stored at -70°C before experiments, using TNF-α and IL-4 ELISA kits (BD Biosciences Pharmingen, Franklin Lakes, NJ, USA). Degree of TNF-α and IL-4 release was calculated based on the absorbance read at 450 nm using a microplate reader. Intracellular mRNA expressions of both cytokines were investigated through real-time PCR.

### Nitrite assay

RAW 264.7 cells were suspended in phenol red-free DMEM supplemented with 10% FBS at a density of 3 × 10^5^ cells/mL. One milliliter of cell suspension was seeded per well onto a 24-well plate, and cells were incubated at 37°C in a 5% CO_2_ incubator. After 24 h, cells were washed twice with sterile phosphate-buffered saline (PBS), and then the cell pellet was re-suspended in 800 μL phenol red-free with 1% FBS-DMEM. Each well was treated with eBV diluted 100-fold, and the final concentration of LPS was set to 1 μg/mL after pre-treatment with eBV. Cells were cultured in LPS-free media as a negative control. After incubation for 20 h, duplicate samples of 100 μL supernatants were added to a 96-well plate, and 180 μL Griess reagent was added to each well. After shaking at room temperature, absorbance of each well plate was read at 540 nm wavelength. The standard curve was plotted using sodium nitrite solution as standard, and the nitrite concentration in each sample was calculated based on the curve. The inhibitory effect of eBV on nitrite generation was evaluated compared to LPS-only treatment.

### Cytotoxicity assay (MTT assay)

To determine whether suppression of iNOS by eBV was from significant cytotoxicity, 80 μL of the 5 mg/mL MTT solution was added to each well after nitrite assay to reach a final concentration of 500 μg/mL, and cells were incubated for 4 h in a 37°C, 5% CO_2_ incubator. Following removal of all media, 1 mL DMSO was added to dissolve formazan crystals. Then 200 μL of each sample was added to 96-well plates, and absorbance was read at 570 nm with DMSO as a blank. The percentage of survival (%) was calculated as the absorbance ratio of eBV-treated cells to LPS-only-treated cells.

### Preparation of total cell lysates

RAW 264.7 cells were suspended in 10% FBS-DMEM at a density of 3 × 10^5^ cells/mL. Cells were seeded on a 60 mm dish and incubated at 37°C, 5% CO_2_ for 24 h. After washing twice with sterile PBS, the cells were re-suspended in 10% FBS-DMEM, and then treated with eBV and LPS (1 μg/mL) for the indicated time lengths. Following treatment, cells were washed twice with ice-cold PBS, harvested using a scraper in boiling cell lysis buffer, and placed in boiling water for 5 min. After cooling, the samples were stored at -20°C and thawed immediately before protein quantification and electrophoresis. BCA method was employed for protein quantification.

### Preparation of nuclear extracts

Nuclear fraction was prepared as previously described by Beg et al. [29]. In brief, cells were washed in PBS, centrifuged, and then re-suspended in lysys buffer (10 mM Tris-HCl, pH 8.0, 60 mM KCl, 1 mM EDTA, 1 mM dithiothreitol, 100 μM PMSF, and 0.2% NP-40). After lysis for 5 min on ice, the lysates were again centrifuged at 2,500 rpm at 4°C for 4 min. The nucleus pellet was washed in lysis buffer without NP-40 and re-suspended in a nuclear extract buffer of equal volume (20 mM Tris-HCl, pH 8.0, 420 mM NaCl, 1.5 mM MgCl_2_, 0.2 mM EDTA, and 25% glycerol). After lysis for 10 min on ice, the nuclear lysates were vortexed and centrifuged at 14,000 rpm for 5 min. The supernatant was collected and later assayed as the nuclear extract. Protein concentration was determined by the Bradford assay.

### Reporter gene (SEAP; secreted embryonic alkaline phosphatase) assay

Reporter gene assay was performed as previously described by Moon et al. [30] with some modifications to determine the effect of eBV on NF-κB activation. Cells were pre-treated with eBV for 30 min and stimulated with LPS for 16 h afterward. The culture supernatant was heated at 65°C for 5 min and then reacted with SEAP assay buffer [2 M diethanolamine, 1 mM MgCl2, 500 μM 4-methylumbelliferyl phosphate (MUP)] in the dark at 37°C for 1 h. Fluorescence from the SEAP/MUP reaction was read by a 96-well plate fluorometer with excitation at 360 nm and emission at 449 nm. The measurement was normalized to protein concentration. Data are expressed in relative fluorescence units compared to that of the vehicle only-treated control.

### Western blot analysis

Thirty to fifty micrograms of protein samples were electrophoresed in 8 ~ 10% SDS-polyacrylamide gel at 100 V for 2 h. The specific portion of interest was cut from the gel, and contained proteins were transferred to a polyvinylidene difluoride (PVDF) membrane (Millipore, Bedford, MA, USA). The membrane was washed twice with PBST (PBS with 0.1% Tween-20) and blocked in blocking buffer with 5% non-fat dry milk at room temperature for 1 h. Afterwards, the membrane was washed with PBST 3 times for 5 min each and then incubated with primary antibodies (iNOS, COX-2, TNF-α, IL-1β, p65, p-p65, IκB-α, STAT1, p-STAT1, STAT3, p-STAT3, IRF3, p-IRF3, and β-actin) and diluted at 1:1,000 to 1:2,000 in 2.5% BSA-PBST at 4°C for 12 h. Later, the membrane was washed again 3 times with PBST for 5 min each and incubated with the corresponding HRP-conjugated secondary antibodies diluted at 1:1,500 to 1:2,000 at room temperature for 2 h. Finally, after washing with PBST 3 times for 5 min each, the membrane was reacted with ECL Western blotting detection reagents (LabFrontier, Suwon, Korea) to emit luminescence, which was measured using LAS 4000 (Fuji Film Corp., Tokyo, Japan).

### Real-time polymerase chain reaction (Real-time PCR)

RAW 264.7 cells were suspended in 10% FBS-DMEM at a density of 3 × 10^5^ cells/mL, seeded on a 60 mm dish, and then incubated in 37°C, 5% CO_2_ for 24 h. The cells were then washed twice with sterile PBS and re-suspended in serum-free DMEM. Cells were treated with 1 μg/mL LPS for 4 h after 1 h of pre-treatment with eBV. RNA was isolated as reported by Chomczynski and Sacchi [31]. In brief, after washing the dish twice with PBS, the cells were lysed with 1 mL of TRI agent (Invitrogen, Carlsbad, CA, USA), and chloroform was added to isolate RNA from the cells. To precipitate the RNA, isopropanol was added to the lysate, and following washing with 75% ethanol, the precipitated RNA pellet was dissolved in nuclease-free water. To increase solubility, the dissolved RNA was heated at 55°C for 10 min and then immediately placed on ice for at least 5 min so that the RNA could be assumed to be in the single-stranded form. Purity was examined by reading the absorbance at 260 nm for RNA and at 280 nm for protein.

One microgram of RNA sample was used for reverse transcription, which was performed via the reverse transcriptase of the avian myelobastosis virus (AMV) (Promega, Madison, IW, USA) and oligo dT primer at 42°C for 60 min. The mix was heated at 99°C for 5 min and then placed on ice to inactivate the reverse transcriptase and to ensure the generated cDNA remained as linear strands. Five microliters of cDNA, diluted 50-fold, was added to both the iQ^™^ SYBR^®^ Green Supermix (Bio-Rad, Hercules, CA, USA) and the primers (indicated in Table 1) to achieve a total volume of 20 μL. Real-time PCR of the mix was performed using a MiniOpticon system (Bio-Rad, Hercules, CA, USA). The threshold cycle (C_T_) value was used to indicate the number of cycles when the amplified amount of target gene reaches a threshold. The C_T_ value of each sample was calculated according to comparative C_T_ method (ΔΔ C_T_ method) using MJ Opticon Monitor software (Bio-Rad, Hercules, CA, USA).

**Table 1:** Sequences of target gene-specific primers used in real-time PCR.

	Target genes		Sequences
Real-time PCR	IL-4 (rat)	Sense	5′- ACC TTGCTGTCACCCTGTTC-3′
		Antisense	5′- TTGTGAGCGTGGACTCATTC-3′
	TNF-α (rat)	Sense	5′- CAAGGAGGAGAAGTT CCCAA-3′
		Antisense	5′- CGGACTCCGTGATGTCTAAG-3′
	β-actin (rat)	Sense	5′- CCCATACCCACCATCACACC-3′
		Antisense	5′- CACCCGCGAGTA- CAACCTTC-3′
	iNOS (mouse)	Sense	5′- GGAGCGAGTTGTGGATTGTC-3′
		Antisense	5′- GTGAGGGCTTGGCTGAGTGAG-3′
	COX-2 (mouse)	Sense	5′- GAAGTCTTTGGTCTGGTGCCTG-3′
		Antisense	5′- GTCTGCTGGTTTGGAATAGTTGC-3′
	TNF-α (mouse)	Sense	5′- CTGTAGCCCACGTCGTAGC-3′
		Antisense	5′- TTGAGATCCATGCCGTTG-3′
	IL-1β (mouse)	Sense	5′- AGTTGACGGACCCCAAAAG-3′
		Antisense	5′- AGCTGGATGCTCTCATCAGG-3′
	IFN-β (mouse)	Sense	5′- CACAGCCCTCTCCATCAACTA-3′
		Antisense	5′-CATTTCCGAATGTTCGTCCT-3′
	β-actin (mouse)	Sense	5′- AGACTTCGAGCAGGAGATGG-3′
		Antisense	5′- ACCGCTCGTTGCCAATAGT-3′

### Animals

Male Sprague Dawley (SD) rats (150 ~ 170 g, 5 weeks old) were purchased from Joong-Ang Laboratory Animals, Inc. (Seoul, Korea). The rats were provided with food pellets and water *ad libitum* under standard experimental conditions. The environment was maintained at 22 ± 2°C, and put under an auto-controlled light/dark cycle for 12 h each. The rats were adapted to the environment for one week prior to experiments. All animal experiments were carried out in accordance with the U.K. Animals (Scientific Procedures) Act, 1986 and associated guidelines, the European Communities Council Directive of 24 November 1986 (86/609/EEC) and the National Institutes of Health guide for the care and use of Laboratory animals (NIH Publications No. 8023, revised 1978); all animal subjects were euthanized under CO_2_ gas according to the guidelines. This study has been approved by the Institutional Animal Care and Use Committee of Seoul National University (permission number: SNU-140117-2).

### Carrageenan-induced paw edema

To examine anti-inflammatory activity of eBV *in vivo*, a carrageenan-induced acute paw edema model was used in SD rats [32]. Single subcutaneous injections of eBV dissolved in sterile water were administered to the hindlimb at doses of either 0.5 or 1 mg/kg [33]. Diclofenac was used as positive control and administered once at a dose of 5 mg/kg to the control group. Thirty min after administration, paw edema was induced by sub-plantar injection of 0.1 mL of 1% carrageenan. The paw volume was measured using a plethysmometer (Ugo Basile, Comerio, Milan, Italy) immediately and at 30 min, and 1, 2, 3, 4, 5, 6, 8, and 24 h after injection.

### Determination of production of pro-inflammatory mediators in inflamed tissue

The edematous tissue of rats induced by carrageenan was harvested, and protein samples were isolated using a protein extract kit (Active Motif, Carlsbad, CA, USA). With the lysates, expression of inflammatory enzymes (iNOS and COX-2) and pro-inflammatory cytokines (TNF-α and IL-1β) were investigated through Western blot.

### Measurement of PGE2, TNF-α, and IL-1β levels in serum

Blood samples from the SD rats were collected from the rats’ hearts after sacrificing, centrifuged at 1,500 rpm for 10 min, and then stored at -70°C for later analyses. The levels of prostaglandin E_2_ (PGE_2_), TNF-α and IL-1β were measured through immunosorbent assay method using ELISA kits (R&D Systems, Minneapolis, MN, USA). All analyses were performed according to the manufacturer’s instructions.

### Statistical analysis

**Fig 1:**
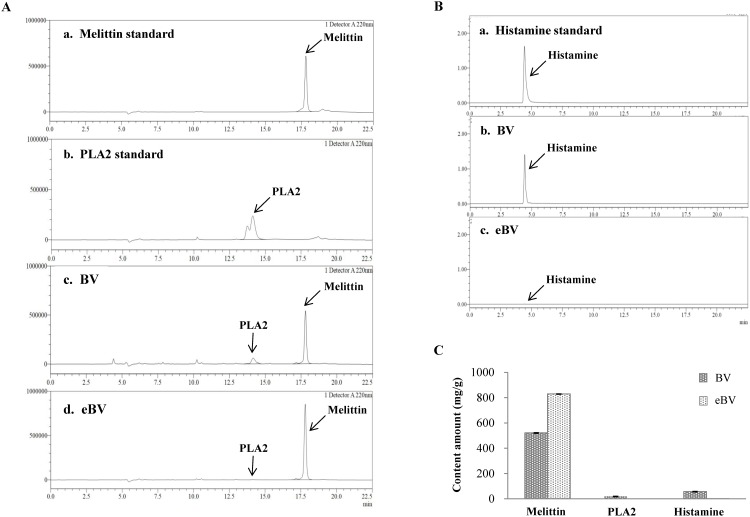
HPLC chromatograms and amount of melittin, PLA2 and histamine. (A) HPLC chromatograms of melittin (tR 17.8 min), PLA2 (tR 14.2 min) and (B) histamine (tR 4.58 min) (C) The amount of melittin, PLA2, and histamine in BV and eBV.

**Fig 2:**
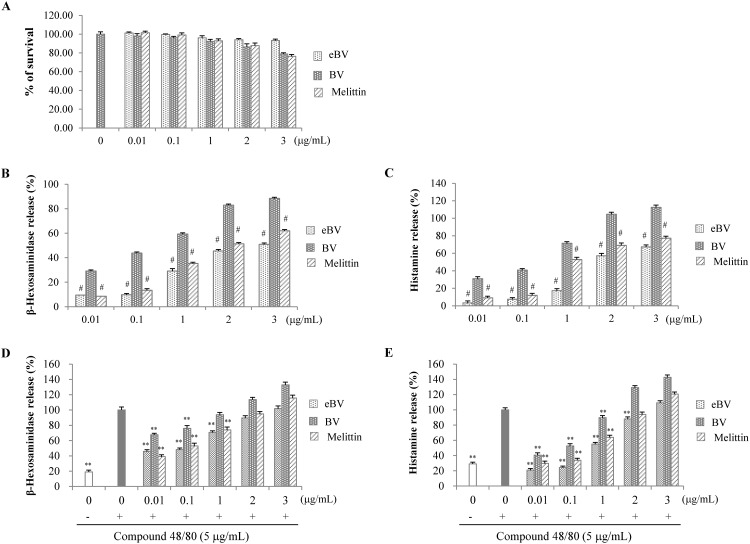
Effect of eBV on β-hexosaminidase and histamine release in compound 48/80-stimulated RBL-2H3 cells. (A) Cell viability was measured via the MTT method as described in the Materials and Methods. (B, C) RBL-2H3 cells were treated with eBV, BV, and melittin (0.01 ~ 3 μg/mL) for 30 min. (D, E) RBL-2H3 cells were treated with compound 48/80 (5 μg/mL) in the presence of eBV, BV, and melittin (0.01 ~ 3 μg/mL) for 30 min. Release of β-hexosaminidase was measured at 405 nm. The release of histamine in RBL-2H3 cells was detected using LC/MS system. Data are presented as mean ± SD (n = 3). ^#^*P* < 0.01 vs. BV group; **P < 0.01 indicates statistically significant difference from the compound 48/80 group.

**Fig 3:**
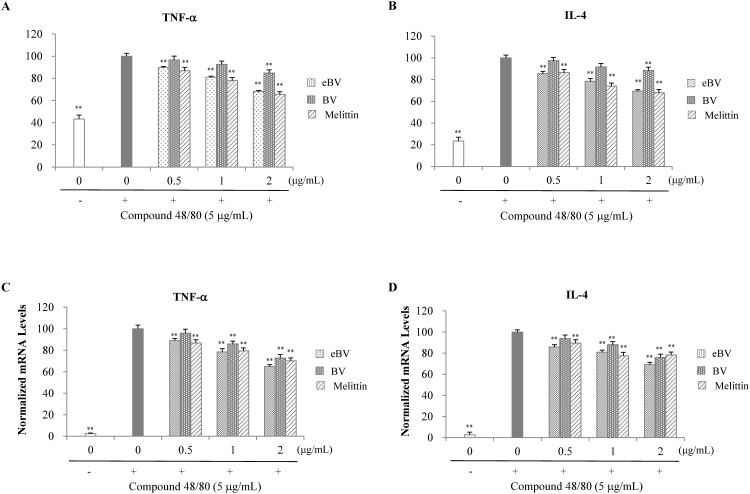
Effect of eBV on TNF-α and IL-4 in compound 48/80-stimulated RBL-2H3 cells. (A, B) RBL-2H3 cells were treated with compound 48/80 (5 μg/mL) in the presence of eBV, BV, and melittin (0.5, 1, and 2 μg/mL) for 30 min. TNF-α and IL-1β levels were measured using an ELISA kit. (C, D) RBL-2H3 cells were treated with compound 48/80 (5 μg/mL) in the presence of eBV, BV, and melittin (0.5, 1, and 2 μg/mL) for 30 min. Total RNA was isolated and further analyzed by real-time PCR. The results are presented as relative expression levels compared with those in unstimulated cells and were normalized to β-actin expression. Data are presented as mean ± SD (n = 3). **P < 0.01 indicates statistically significant difference from the compound 48/80 group.

**Fig 4:**
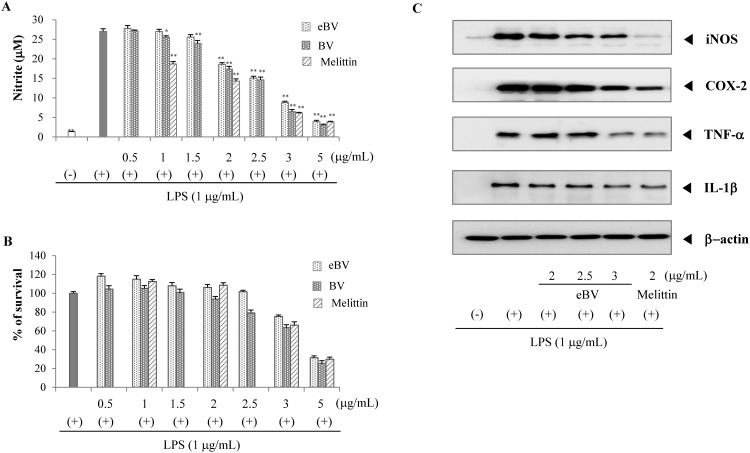
Effect of eBV on LPS-induced NO production and protein expression in macrophage cells. (A) RAW 264.7 cells were stimulated with LPS (1 μg/mL) in the presence or absence of eBV. After 20 h, the cultured media were collected, and nitrite concentration was analyzed using Griess reaction. The data are expressed as mean ± SD of triplicate tests. *p < 0.05 and **p < 0.01 indicate statistically significant difference compared with the LPS (+) group. (B) Cell viability was measured by the MTT method as described in the Materials and Methods. (C) The effect of eBV on iNOS, COX-2, TNF-α, and IL-1β protein expression in LPS-stimulated RAW 264.7 cells. RAW 264.7 cells (5 × 10^5^ cells/mL) were incubated for 24 h and then treated with LPS (1 μg/mL) and eBV for an additional 4 h and 20 h. After incubation, total cell extracts were obtained and subjected to Western blot analysis as described in the Materials and Methods. The data are representative of three separate experiments. β-actin was used as an internal standard.

**Fig 5:**
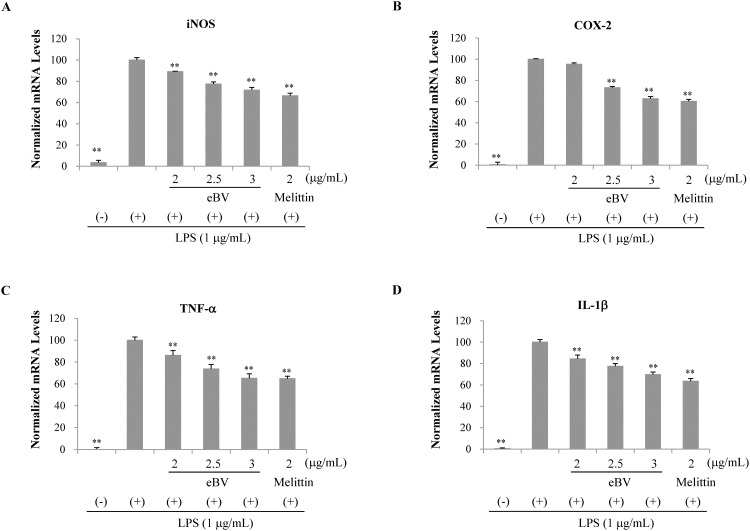
Effect of eBV on LPS-induced iNOS, COX-2, TNF-α, and IL-1x mRNA expression in macrophage cells. RAW 264.7 cells were treated with LPS (1 μg/mL) and eBV for 4 h. Total RNA was isolated and further analyzed by real-time PCR. The results are presented as relative expression levels compared with those in unstimulated cells and were normalized to β-actin expression. Data are presented as mean ± SD (n = 3). **P < 0.01 indicates statistically significant difference compared with the LPS (+) group.

**Fig 6:**
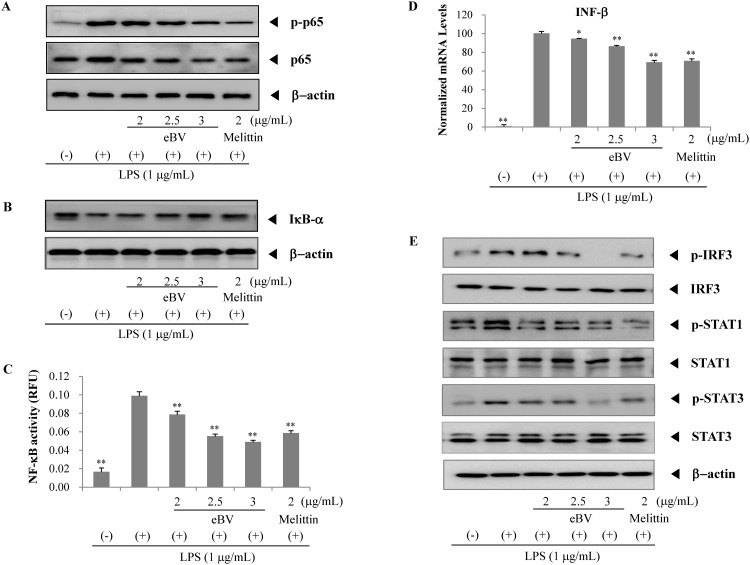
Effect of eBV on the TRIF pathway in LPS-induced macrophage cells. (A, B) Effect of eBV on p65, p-p65 and IkB-α protein expression in LPS-stimulated RAW 264.7 cells. RAW 264.7 cells were incubated for 24 h and then treated with LPS (1 μg/mL) and eBV for an additional 4 h. After incubation, nuclear and total cell extracts were obtained and subjected to Western blot analysis as described in the Materials and Methods. (C) SEAP-RAW cells were stimulated with LPS (1 μg/mL) in the presence or absence of eBV. After 16 h, the supernatants were analyzed for determination of SEAP activity. Values are expressed as mean ± SD (n = 3). (D) RAW 264.7 cells were treated with LPS (1 μg/mL) and eBV for 4 h. Total RNA was isolated and further analyzed by real-time PCR. The results are presented as relative expression levels compared with those in unstimulated cells and were normalized to β-actin expression. Data are presented as mean ± SD (n = 3). *P < 0.05 and **P < 0.01 indicate statistically significant difference compared with the LPS (+) group. (E) Effect of eBV on p-IRF3, IRF3, p-STAT1, STAT1, p-STAT3, and STAT3 protein expression in LPS-stimulated RAW 264.7 cells. Data are representative of three separate experiments. β-actin was used as an internal standard.

**Fig 7:**
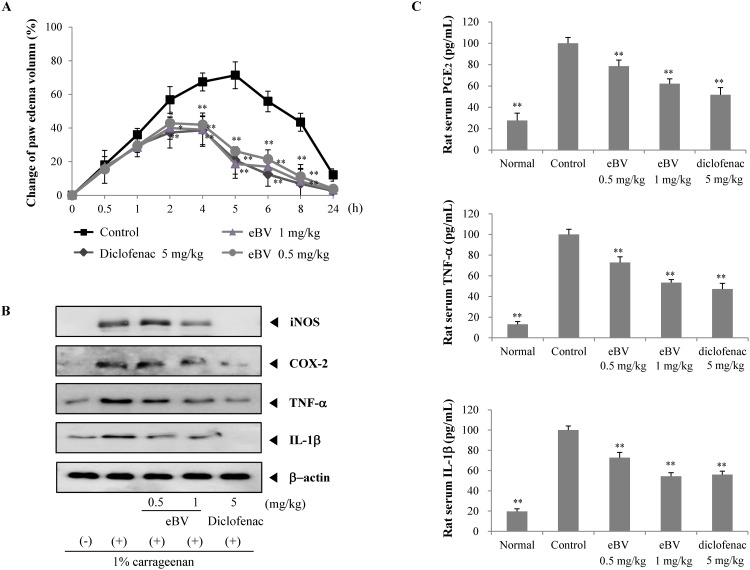
Effect of eBV on carrageenan-induced paw edema. (A) eBV was injected 30 min before carrageenan injection in the right paw of SD rats. The paw volume was measured before (0 h) and at intervals of 0.5, 1, 2, 4, 6, 8, and 24 h after carrageenan injection using a plethysmometer. Data are shown as mean ± SD (n = 6). *P < 0.05 and **P < 0.05 indicate statistically significant difference from the control group. (B) Expression of iNOS, COX-2, TNF-α, and IL-1β in carrageenan-induced paw tissue was determined by Western blot analysis. Data are representative of three separate experiments. β-actin was used as internal standard. (C) PGE2, TNF-α, and IL-1β levels were measured after *in vivo* injection of eBV. Serum was collected from rats with carrageenan-induced paw edema and analyzed for PGE2, TNF-α, and IL-1β levels using ELISA kit. Data are presented as mean ± SD (n = 6). **P < 0.01 indicates statistically significant difference from the control group.

All experiments were independently performed three times, and the most representative result is shown. Where appropriate, data are presented as mean ± standard deviation (SD). Mean difference between the eBV-treated group and control group was analyzed by one-way analysis of variance (ANOVA); *p* values less than 0.05 were considered statistically significant (*; *p*<0.05, **; *p* < 0.01). See S1 File for the raw data underlying Figs 1–7.

## Results

### Determination of melittin, PLA2, and histamine

Fig 1 shows the HPLC graphs of eBV and BV. According to the HPLC analyses, melittin, PLA, and histamine peaks were observed at 17.8, 14.2, and 4.58 min, respectively. Elimination of PLA2 and histamine, known allergens of BV, was verified in the chromatogram. The peak of melittin is present both in original BV and eBV, whereas the peaks of histamine and PLA2 are only detected in original BV. Moreover, concentration of melittin increased in purified eBV (828.64 mg/g) compared to the original BV (520.64 mg/g).

### Suppression of β-hexosaminidase and histamine degranulation in compound 48/80-stimulated RBL-2H3 cells

To investigate amount of allergic reaction caused by refined eBV removed of allergens compared to original BV, levels of β-hexosaminidase and histamine—indicators of degranulation—were measured in compound 48/80-stimulated RBL-2H3 cells, as shown in Fig 2.

MTT assay confirmed that eBV did not exert significant cytotoxicity at concentrations up to 3 μg/mL. eBV, BV, and melittin increased release of β-hexosaminidase and histamine dose-dependently. Allergen-removed eBV significantly suppressed release of β-hexosaminidase and histamine compared to original BV at the same concentrations (*p* < 0.01). Also, the suppressive effect of eBV on β-hexosaminidase and histamine at concentrations higher than 1 μg/mL was greater than that of melittin. The anti-allergic effect of eBV was examined in relation to compound 48/80, a de-granulating factor. While compound 48/80 stimulation led to significant increase of β-hexosaminidase and histamine release (p < 0.01), eBV suppressed compound 48/80-induced β-hexosaminidase and histamine release at 0.01 ~ 1 μg/mL (p < 0.01). When compared at same concentrations, eBV exhibited greater anti-allergic effect than BV.

### Suppression of proinflammatory cytokine expression in compound 48/80-stimulated RBL-2H3 cells

Proinflammatory cytokines such as histamine, leukotriene, IL-3, IL-4, IL-5, and TNF-α are synthesized and/or released when macrophages are degranulated. Consequently, the cytokines induce up-regulation of IgE antibodies and differentiation of inflammatory cells, causing immediate or early allergic response. Secretion levels of IL-4 and TNF-α from RBL-2H3 cells were measured using ELISA kits (BD Biosciences Pharmingen, Franklin Lakes, NJ, USA), and mRNA expression was analyzed by real-time PCR.

Secretion of TNF-α and IL-4 was significantly enhanced when cells were stimulated with compound 48/80 (*p* < 0.01). However, pre-treatment with eBV significantly inhibited compound-48/80-induced secretion of TNF-α and IL-4 in a dose-dependent manner (*p* < 0.01; Fig 3). Similarly, pre-treatment with eBV also significantly suppressed compound 48/80-induced mRNA expression of TNF-α and IL-4 dose-dependently (*p* < 0.01; Fig 3). The suppressive effect of allergen-removed eBV on release and mRNA expression of IL-4 and TNF-α at the same concentrations was greater than that of BV. The current findings indicate that eBV inhibits allergic response by suppressing release and mRNA expression of IL-4 and TNF-α, as demonstrated in compound 48/80-stimulated RBL-2H3 cells.

### Supression of NO production in LPS-stimulated RAW 264.7 cells

NO is a major mediator of inflammatory responses, mostly generated by iNOS. The enzyme iNOS is closely related with inflammatory reaction and carcinogenesis; NO generated by iNOS was shown to induce expression and activation of COX-2 [34]. In light of these findings, the suppressive effect of eBV on LPS-induced NO generation was examined with regard to its anti-inflammatory effect.

NO generation was found to increase significantly compared to the vehicle-treated control when RAW 264.7 cells were stimulated with LPS (p < 0.01). However, eBV treatment suppressed LPS-induced generation of NO in a dose-dependent manner (p < 0.01; Fig 4). MTT assay found that cell viability was maintained at >75% up to 3 μg/mL concentrations of eBV. This data confirms that the decrease in NO generation from eBV treatment is not due to cytotoxicity (Fig 4).

### Suppression of iNOS, COX-2, TNF-α, and IL-1β expression in LPS-stimulated RAW 264.7 cells

To clarify whether the inhibitive effect of eBV on NO generation is related to modulation of the iNOS enzyme, the mRNA and protein expressions of iNOS were examined using real-time PCR and Western blot analyses, respectively. After pre-treatment with eBV, cells were stimulated with LPS for activation, and the lysates were collected for experimentation. eBV was found to suppress mRNA and protein expression of iNOS in a dose-dependent manner (Figs 4 and 5).

Like iNOS, COX-2 is a well-known mediator of inflammatory response, the expression of which is regulated by NF-κB transcription factor. As COX-2 is closely related to expression of iNOS, the mRNA and protein expression levels of COX-2 were investigated, and eBV was shown to inhibit both mRNA and protein expression of COX-2 dose-dependently (Figs 4 and 5).

The cytokine TNF-α is up-regulated when inflammatory cells are activated by stimulants such as LPS. Expression of TNF-α plays a crucial role in mediating expression of IL-1β and progression of inflammation. IL-1β is also an important factor in inflammation as IL-1β relates to various gene expressions regarding inflammation and tissue injury. mRNA and protein expression of TNF-α and IL-1β were therefore studied. While all mRNA and protein expressions of TNF-α and IL-1β increased when RAW 264.7 cells were stimulated with LPS, pre-treatment with eBV suppressed increase in a dose-dependent manner (Figs 4 and 5). Thus, eBV was found to possess anti-inflammatory properties, as eBV suppressed the mRNA and protein expression of various inflammatory mediators.

### Suppression of NF-κB in LPS-stimulated RAW 264.7 cells

NF-κB is a transcription factor consisting of p65/p50 subunits and stays inactive in the cytoplasm, bound to IκB when unstimulated. When stimulated (e.g. by LPS), NF-κB becomes active through degradation of IκB, which is the consequence of phosphorylation by IκB kinase (IKK). In a free state, NF-κB translocates to the nuclei, binds to NF-κB-binding promoters, and regulates expression of pro-inflammatory genes such as iNOS, COX-2, TNF-α, and IL-1β [35].

To further examine the effect of eBV on activation of NF-κB, reporter gene assay was performed. NF-κB transcriptional activity is associated with the level of NF-κB subunits p65 in the nucleus, and was detected using Western blot analysis. eBV was found to suppress LPS-induced protein expression of nuclear p65 and p-p65 in a dose-dependent manner, and to also suppress LPS-induced degradation of IκB dose-dependently. In the reporter gene assay for NF-κB transcriptional activity (SEAP), stimulation of RAW 264.7 cells with LPS elicited a 6-fold increase in NF-κB transcriptional activity, but concurrent treatment with eBV effectively inhibited the LPS stimulated increase of NF-κB transcriptional activity (Fig 6).

### Suppression of phosphorylation of IRF3 and STAT1 in LPS-stimulated RAW 264.7 cells

When phosphorylated, IRF3 becomes activated in the TRIF-dependent pathway. Active IRF3 translocates into the nucleus, binding to the promoter region and activating transcription of type 1 INF. In turn, type 1 INF phosphorylates and activates STAT1. Phosphorylated STAT1 acts as a transcription factor for inflammatory mediator expression [26, 27]. As shown in Fig 6, eBV significantly inhibited mRNA expression of IFN-β in LPS-stimulated RAW 264.7 cells dose-dependently (*p* < 0.01). Moreover, eBV was found to suppress phosphorylation of STAT1, which is induced by IFN-β, in a dose-dependent manner, and eBV also suppressed phosphorylation of IRF3 dose-dependently. The effect of eBV on activation of STAT3, a major inflammatory transcription factor, was also investigated. Phosphorylation of STAT3 was decreased by eBV in a dose-dependent manner. Based on these findings, the underlying mechanism for the anti-inflammatory potential of eBV is surmised to involve modulation of the TRIF-dependent pathway.

### Inhibition of carrageenan-induced paw edema in rats

To further investigate the anti-inflammatory activity of eBV *in vivo*, a carrageenan-induced paw edema model was employed. Acute edema was induced through a single, sub-plantar injection of carrageenan (0.1 mL of 1% solution) to the left paw, the volume of which was measured 24 h later. Volume reached its highest peak at 5 h after injection, and eBV suppressed edema in a dose-dependent manner (Fig 7). Thus, the anti-inflammatory potential of eBV was also verified in an *in vivo* acute inflammatory model.

### Suppression of inflammatory mediator expression in carrageenan-induced paw edema in rats

The *in vivo* effects of eBV on protein expression of inflammatory enzymes (COX-2 and iNOS) and pro-inflammatory cytokines (TNF-α and IL-1β) were investigated from the tissue of sacrificed animals. Protein expression of iNOS, the key enzyme in NO generation, and COX-2, the downstream signaling protein of iNOS, in each animal group were examined through Western blot assay. As shown in Fig 7, while expression of iNOS and COX-2 proteins was higher in the carrageenan-induced edema group compared to the untreated group, expression was down-regulated in the eBV-treated group compared to the carrageenan-only control group.

Protein expression of TNF-α and IL-1β was also investigated in groups using Western blot analysis. Expression of TNF-α and IL-1β in the carrageenan-treated group was higher than in the untreated group, whereas expression was suppressed in the eBV-treated group compared to the carrageenan-only control group.

### Inhibitory effect of eBV on PGE_2_, TNF-α, and IL-1β levels in serum

To further determine the anti-inflammatory effects of eBV *in vivo*, enzyme immunoassay (EIA) was performed on serum PGE2, TNF-α, and IL-1β levels. When acute inflammation was induced by carrageenan injection, serum levels of PGE_2_, TNF-α, and IL-1β in the carrageenan-injected group significantly increased compared to the untreated group (*p* < 0.01). However, levels of PGE_2_, TNF-α, and IL-1β in the eBV-treated group were down-regulated compared to the control group (*p* < 0.01) (Fig 7). In particular, the inhibitive effect of eBV at dose 1 mg/kg on PGE_2_ and TNF-α were stronger than diclofenac at 1 mg/kg, though not statistically significant. The current results imply that eBV possesses potent anti-inflammatory properties in *in vivo* acute inflammation settings and in *ex vivo* tissue and serum samples.

## Discussion

With increasing life expectancy and heightened interest in improving quality of life (QoL), the need for effective treatment and prevention of modern day diseases is growing, with special focus on inflammatory processes as they are known to be closely related with pathologies such as cancer, Alzheimer’s disease, and atherosclerosis. Various treatment alternatives in complementary and alternative medicine are being explored in an attempt to treat such diseases highly correlated with lifestyle [36–38].

Numerous studies on biochemical components, pharmacological activity, underlying mechanisms, hypersensitivity and toxicity of BV have been performed, and although two studies on BV safety reported that stimulating sensations and edematous reactions take up the majority of hypersensitivity reaction cases [39, 40], incidence of anaphylactic shock, albeit rare, must be referred to with caution.

Recently, investigations on allergen-removed BV reported effects on whitening, wrinkle care, and anti-inflammation through suppression of NO generation [41, 42]. Still, there is a paucity of comparative studies on the anti-inflammatory and anti-allergic potential of original BV and allergen-free refined BV, and the current study holds significance in examination of the anti-allergic effect of eBV *in vitro*, and the anti-inflammatory activity of eBV both *in vitro* and *in vivo*.

HPLC analysis was performed with eBV to determine the presence of primary known allergens; BV, PLA2 and histamine. Results demonstrated that PLA2 and histamine concentration decreased in eBV after isolation and purification, which lead to relative increase in concentration of melittin, the main active compound of eBV (Fig 1).

β-Hexosaminidase and histamine are released when mast cells are activated by antigens or immunologic stimulation. They are therefore regarded markers of degranulation in *in vitro* experiments of allergic response [43, 44]. Compound 48/80 is a molecule known to induce degranulation of mast cells during allergic response [45]. In the current results, eBV was found to significantly suppress compound 48/80-induced secretion of β-hexosaminidase and histamine (*p* < 0.01), and the suppressive effect of eBV was greater than that of original BV (Fig 2). Although eBV was prepared to be free of histamine and PLA2, BV contains various allergens. Moreover, melittin itself has been shown to induce allergic reaction and inflammation under certain conditions [46, 47]. Nevertheless, the anti-inflammatory properties of BV and melittin described in the previous literature suggest therapeutic benefit [48, 49] and BV pharmacopuncture is widely used in Korean medicine practice [50, 51]. The authors have also conducted a clinical trial comparing the anti-inflammatory and local adverse effects and allergic reaction to BV and eBV, which is currently submitted to be considered for publication. The current study holds significance in that the anti-allergic activity of allergen-free eBV is greater than that of original BV *in vitro*.

When activated, mast cells express and release inflammatory cytokines, which in turn modulate the inflammatory process. IL-1β activates mast cells through mediation of IgE and induces cells to release histamine and Th2 cytokine, contributing to progression of IgE-mediated allergic inflammation. TNF-α is a pro-inflammatory cytokine that is released during the early phase of immunologic response and is known to be involved in modulation of allergic inflammation [52]. IL-4 potentiates synthesis of IgE and induces immediate type 1 allergic reactions by helping B cells transform to express IgE, and up-regulating IL-5, which is crucial for differentiation of eosinophils [53]. While secretion and mRNA expression of both TNF-α and IL-4 in compound 48/80-stimulated RBL-2H3 cells increased significantly (*p* < 0.01), expression in cells pre-treated with eBV prior to compound 48/80 stimulation were shown to be significantly lower than the control (*p* < 0.01; Fig 3). The inhibitory effect of eBV removed of PLA2 and histamine on allergic response was observed. Therefore, utilization of eBV is expected to be beneficial in terms of fewer clinical adverse effects including pain and swelling.

The role of macrophages in NO generation is, as of yet, controversial [54, 55]. Activation of macrophages in allergic inflammation and the consequent effect on NO as a mediator of inflammation were investigated in the current study. In the iNOS assay, eBV was found to inhibit NO generation in a dose-dependent manner, and MTT assay confirmed that the result was not due to significant cytotoxicity (Fig 4). Additionally, eBV suppressed expression of iNOS and COX-2 dose-dependently. Pro-inflammatory cytokines such as TNF-α and IL-1β are known to be up-regulated during inflammatory response, and expression of TNF-α and IL-1β were also suppressed by eBV in a dose-dependent manner (Figs 4 and 5).

NF-κB is known as the central transcription factor that regulates inflammatory mediators such as iNOS, COX-2, and TNF-α [31]. When inactive, NF-κB stays in the cytoplasm as a heterodimer consisting of p50 and p65 subunits, bound to IκB, the inhibitor of NF-κB protein. When NF-κB is activated by stimulants such as LPS, phosphorylation and degradation of IκB allows NF-κB to become active and to translocate into the nuclei, subsequently acting as a transcription factor. Results from Western blot analysis revealed that eBV suppressed degradation of IκB and intra-nuclear translocation of p-p65. eBV also inhibited the LPS-stimulated increase of NF-κB transcriptional activity in a dose dependent manner (Fig 6).

In addition, the inhibitory effect of eBV on NF-κB-mediated transcription was examined in regard to the TRIF-dependent pathway. As shown in the current findings, eBV inhibited phosphorylation of IRF3, the downstream signal of the TRIF-dependent pathway, leading to down-regulated expression of IFN-β, the transcription of which is initiated by phosphorylated IRF3. Furthermore, phosphorylation of STAT1 and STAT3, the sequel of IFN-β, was also suppressed by eBV in a dose-dependent manner (Fig 6). The above findings illustrate that eBV, eliminated of allergens, may act as a potent inhibitor of various inflammatory mediators.

Acute inflammation is accompanied by characteristic symptoms such as edema, pain, and loss of function. Edematous change is one of the primary indicators of inflammatory response [56]. A carrageenan-induced acute paw edema model was studied to the aim of further exploring the anti-inflammatory potential of eBV *in vivo*. As shown in the study outcome, eBV suppressed carrageenan-induced paw edema dose-dependently. Additionally, edematous paw tissue was investigated for various inflammatory proteins. eBV was found to significantly suppress carrageenan-induced increase in proteins such as iNOS, COX-2, TNF-α, and IL-1β in local tissue. Moreover, the serum levels of PGE2, TNF-α, and IL-1β were down-regulated by eBV treatment (Fig 7). These results imply that eBV exerts potent anti-inflammatory effects both *in vivo* and *in vitro*.

In conclusion, anti-allergic and anti-inflammatory effects of eBV were demonstrated using *in vitro* and *in vivo* allergic inflammatory models. Allergen-removed eBV was found to exert anti-allergic potential, and it can be conjectured that eBV displays anti-inflammatory effect through regulation of the IRF3 signaling pathway and its downstream factors. Therefore, eBV may be considered a feasible candidate in therapeutic approach to inflammatory disease using complementary and alternative medicine.

## Supporting Information

S1 File Raw data underlying Figs 1–7. The raw data for Figs 1–7 are presented as mean and standard deviation (SD) values.
(XLSX)
